# Supplementary figures and images for: Geospatial HIV-1 subtype C gp120 sequence diversity and its predicted impact on broadly neutralizing antibody sensitivity
Source: PLoS One. 2021 May 24;16(5):e0251969. doi: 10.1371/journal.pone.0251969 (PMC8143386; doi:10.1371/journal.pone.0251969)

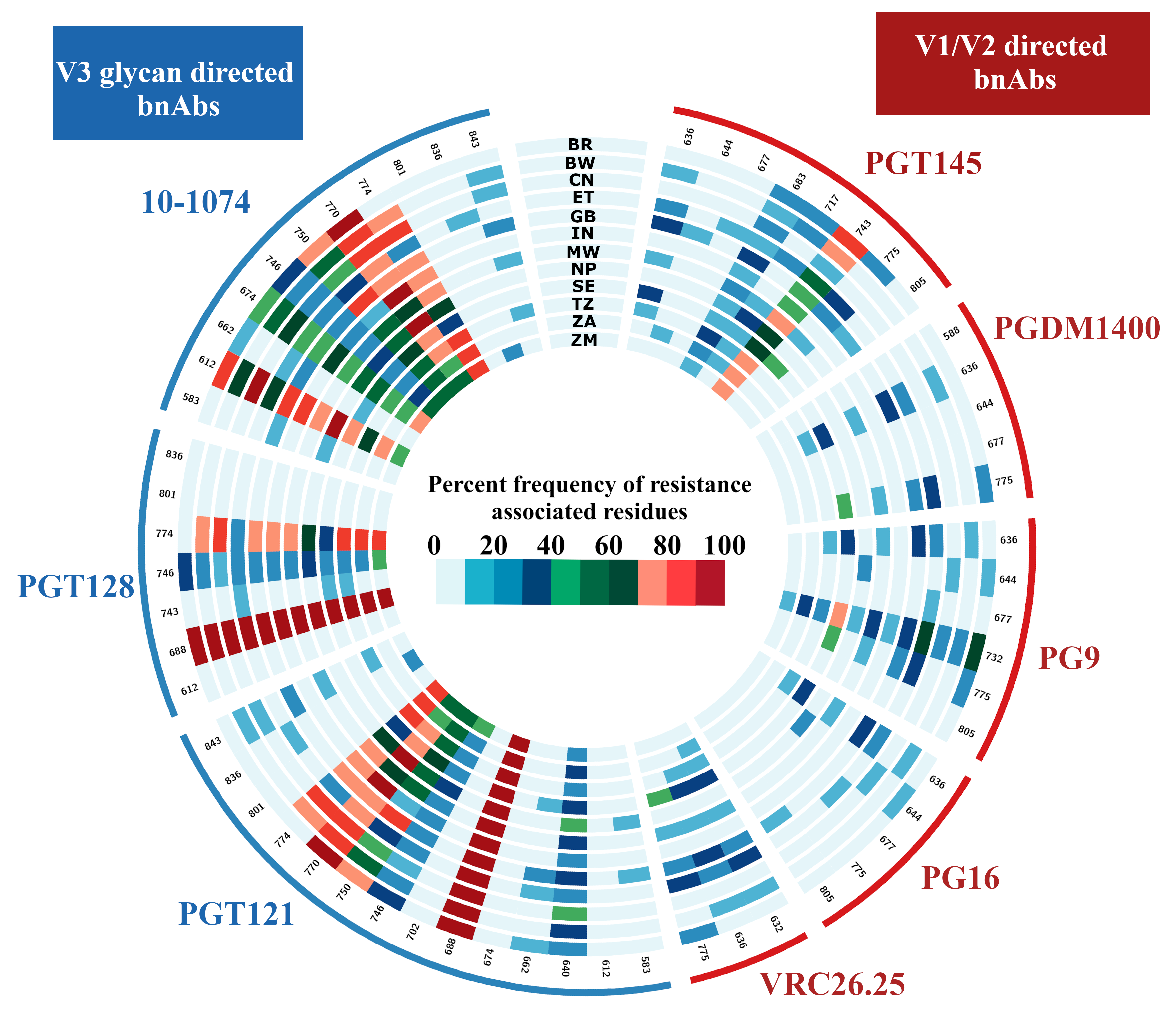

Supplement: S1 Fig — A circos heatmap was plotted based on analysis of 1768 sequences from the HIV database (www.hiv.lanl.gov) to depict the abundance of known amino acid residues associated with resistance to the following bnAbs: PGT145, PGDM1400, PG9, PG16, CAP256-VRC26.25, PGT121, PGT128 and 10–1074. Each track indicates the country of origin and each pixel on the circular track indicates position of specific residue associated with resistance to indicated bnAbs. (TIF) [file pone.0251969.s001.tif]
